# Supplementary material for: MetaRibo-Seq measures translation in microbiomes
Source: Nat Commun. 2020 Jun 29;11:3268. doi: 10.1038/s41467-020-17081-z (PMC7324362; doi:10.1038/s41467-020-17081-z)
Supplement: Supplementary file 10 — Supplementary Data 7 [file 41467_2020_17081_MOESM10_ESM.zip › File2/Confidence_High_Taxonomy/200750_out.krona.html]

Javascript must be enabled to view this page.

members
magnitude
magnitudeUnassigned
count
unassigned
taxon
rank

200750\_out

14

2
superkingdom
14

14
976
phylum

14
class
200643

14
order
171549

14
family
171552

genus
838
14

1

SRS078176\_contig\_number\_16150
species
2293125

165179
species

SRS012849\_contig\_number\_27647SRS018888\_contig\_number\_9749SRS044535\_contig\_number\_15029SRS049712\_contig\_number\_8592SRS049995\_contig\_number\_36513SRS053398\_contig\_number\_22640SRS103987\_contig\_number\_1833SRS104036\_contig\_number\_3717SRS105082\_contig\_number\_10969SRS143181\_contig\_number\_contig-100\_673.78009SRS143876\_contig\_number\_19940SRS144506\_contig\_number\_31393SRS144603\_contig\_number\_11385
13
